# A Brief Coaching Pilot Enhances Professional Identity Formation and Clinical Skills Acquisition During Emergency Medicine Clerkships Shortened by COVID-19

**DOI:** 10.5811/westjem.2021.12.53917

**Published:** 2022-01-01

**Authors:** William Dixon, Moises Gallegos, Sarah Williams

**Affiliations:** Stanford University School of Medicine, Department of Emergency Medicine, Palo Alto, California

## Abstract

**Introduction:**

The Covid-19 pandemic limited educational and career development opportunities for medical students, requiring innovative programs to accelerate professional identity formation and clinical skills acquisition.

**Methods:**

We developed a brief coaching intervention that took place over the advanced (sub-internship) emergency medicine rotation at our institution. We trained coaches using a newly developed workshop, who met with students for an average of 4.5 hours over 3 weeks.

**Impact/effectiveness:**

We showed that this coaching program was both feasible and impactful for faculty coaches and medical students.

## BACKGROUND

The COVID-19 pandemic has been disruptive to medical education, curtailing clinical experiences vital for training and professional identity formation. At our institution, multiple student clerkships were delayed while safety measures were instated. “Away” audition rotations were cancelled. Clerkships such as our advanced emergency medicine (EM) rotation were shortened to three weeks, further limiting potential preparation for residency.

As clerkship directors and advisors, we were concerned that students would have diminished opportunities to acquire clinical skills within the speciality and have difficulty developing their professional identity. Professional identity formation is a key outcome of undergraduate medical education (UME). Medical students take their personal identity and values, combine inputs from role models and clinical experiences, and determine if their professional identity is the right “fit”in a chosen specialty.^1^,^2^

To address these challenges, we looked to foster meaningful and authentic partnerships between medical students and EM faculty through the development and implementation of a brief coaching intervention. Coaching can enhance skills training and performance, motivation, and well-being.^3^,^4^,^5^,^6^ However, coaching interventions often take place over months.^7^ Effectiveness regarding skill acquisition and professional identity formation in coaching for medical education has not been shown over such a brief period as three weeks.

## OBJECTIVES

We aimed to implement an impactful coaching pilot over our 3-week advanced EM clerkship shortened by COVID. Our objectives were to enhance clinical skills acquisition and provide a trusted relationship for conscious reflection around professional identity formation.

## CURRICULAR DESIGN

The two most relevant conceptual frameworks related to our coaching intervention are professional identity formation^1^,^2^ and Kolb’s experiential learning cycle.^8^ A crucial outcome of advanced clerkships is to transcend clinical knowledge acquisition and place the student in an experiential environment with direct responsibility for clinical decision making and patient care.^2^ Coaching facilitates professional identity formation via experiential learning, allowing students to take a concrete experience and participate in reflective observation, subsequent abstract conceptualization, and then plan for active experimentation for their next shift.^8^,^9^ Our curriculum development process was informed by Kern et al.^10^ This project was reviewed and cleared by the IRB/Research Compliance office at Stanford University. We recruited a coach cohort of five faculty with an interest in medical education. To protect psychological safety, we identified junior faculty who were *not* part of residency or clerkship leadership to limit student concern for bias in the subsequent application cycle and encourage candid discussions. We created a virtual two-hour coach training workshop (addendum), reflective of Deiorio and Hammoud’s coaching approach, which included a coaching toolkit.^11^ We invited all advanced EM clerkship students from 6/29/2020 through 8/30/2020 to participate. Eight students accepted, reflecting all students from our home institution applying in EM during the 2020–2021 recruitment cycle.

Students completed a coaching worksheet centered on clerkship and career goals, and identified areas of strength, growth, and specific objectives. Students and coaches met 3–5 times during the 3-week clerkship, facilitating experiential learning. Sessions included goal formation, strength assessments, check-ins, revisiting action plans, and reflective practice on clinical challenges.

## IMPACT/EFFECTIVENESS

Our coaching intervention was rapidly deployed to all EM advanced clerkship students over 3 rotations despite the limitations presented by COVID-19. Impact was measured through an anonymous survey tool with Likert and open-ended items. 15/16 (93.75%) of surveys were completed: 7 student and 8 coach experiences. Our intervention was successful in multiple domains, including:

- Psychological safety: all students were comfortable that coaching discussions would not be used as part of their assessment. All felt they could be candid with their coach in discussing areas of clinical growth.
- Professional identity formation: all students indicated that the coaching program increased their understanding of the field of EM, separately from the clerkship itself. 71.4% indicated improvement of their understanding of whether EM fits with their strengths and values.
- Clinical skills: all students reported that coaching improved their clinical abilities (e.g. development of differential, determination of assessment and plan) during their rotation. All felt more prepared to enter residency.
- All faculty noted that being a coach contributed to their own professional fulfillment and felt prepared to be coaches despite the short training period. Please see the Figure for our results summary.

One example quote from a student: “*My coach helped me immensely during my sub-I. S/he listened to my concerns, validated my experience, provided a different perspective, gave great professional and personal advice, and overall made my experience 1000% better! It was so beneficial to have an “insider” to speak to and bounce ideas off of. S/he helped me gain confidence and enjoy each shift to the fullest*.”

In summary, this was a feasible and impactful intervention. Each coach had a maximum of 2 students. Coaching time averaged 4.5 coaching hours for each student over three weeks. Coaches were volunteer faculty, limiting costs and allowing representation of different subspecialties within the field of EM. Our just-in-time coach training workshop and materials helped position our program to have this impact despite none of our junior faculty coaches having prior coaching experience. Our subjects found the intervention acceptable and impactful. There is significant potential for replication in other specialties or other EM clerkships.

## Figures and Tables

**Figure 1:**
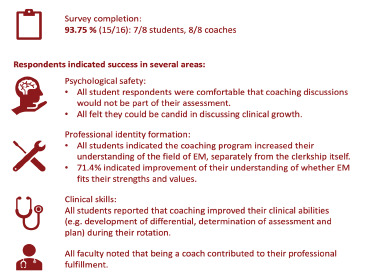
Summary of brief coaching pilot results. *EM*, emergency medicine.
